# Short-Term and Long-Term Outcomes in Mid and Low Rectal Cancer With Robotic Surgery

**DOI:** 10.3389/fonc.2021.603073

**Published:** 2021-03-09

**Authors:** Jingwen Chen, Zhiyuan Zhang, Wenju Chang, Tuo Yi, Qingyang Feng, Dexiang Zhu, Guodong He, Ye Wei

**Affiliations:** Department of General Surgery, Zhongshan Hospital, Fudan University, Shanghai, China

**Keywords:** rectal cancer, robotic surgery, complications, anastomotic leakage, long-term outcomes

## Abstract

**Objective:**

To investigate the risk factors for postoperative complications and anastomotic leakage after robotic surgery for mid and low rectal cancer and their influence on long-term outcomes.

**Methods:**

A total of 641 patients who underwent radical mid and low rectal cancer robotic surgery at Zhongshan Hospital Fudan University from January 2014 to December 2018 were enrolled in this study. The clinicopathological factors of the patients were collected. The risk factors for short-term outcomes of complications and anastomotic leakage were analyzed, and their influences on recurrence and overall survival were studied.

**Results:**

Of the 641 patients, 516 (80.5%) underwent AR or LAR procedures, while 125 (19.5%) underwent the NOSES procedure. Only fifteen (2.3%) patients had stoma diversion. One hundred and seventeen patients (17.6%) experienced surgical complications. Anastomotic leakage occurred in 44 patients (6.9%). Eleven patients (1.7%) underwent reoperation within 90 days after surgery. Preoperative radiotherapy did not significantly increase anastomotic leakage in our study (7.4% vs. 6.8%, P = 0.869). The mean postoperative hospital stay was much longer with complication (10.4 vs. 7.1 days, P<0.05) and leakage (12.9 vs. 7.4 days, P < 0.05). Multivariate analysis showed that male sex (OR = 1.855, 95% CI: 1.175–2.923, P < 0.05), tumor distance 5 cm from the anus (OR = 1.563, 95% CI: 1.016–2.404, P < 0.05), and operation time length (OR = 1.563, 95% CI: 1.009–2.421, P < 0.05) were independent risk factors for complications in mid and low rectal cancer patients. The same results for anastomotic leakage: male sex (OR = 2.247, 95% CI: 1.126–4.902, P < 0.05), tumor distance 5 cm from the anus (OR = 2.242, 95% CI: 1.197–4.202, P < 0.05), and operation time length (OR = 2.114, 95% CI: 1.127–3.968, P < 0.05). The 3-year DFS and OS were 82.4% and 92.6% with complication, 88.4% and 94.0% without complication, 88.6% and 93.1% with leakage, and 87.0% and 93.8% without leakage, respectively. The complication and anastomotic leakage showed no significant influences on long-term outcomes.

**Conclusion:**

Being male, having a lower tumor location, and having a prolonged operation time were independent risk factors for complications and anastomotic leakage in mid and low rectal cancer. Complications and anastomotic leakage might have no long-term impact on oncological outcomes for mid and low rectal cancer with robotic surgery.

## Introduction

Colorectal cancer is the world’s fourth most deadly cancer, with almost 900,000 deaths annually (1). Surgical resection remains the mainstay of curative treatment. The optimal oncological operation consists of low anterior resection or abdominoperineal resection with complete total mesorectal excision (TME) based on the tumor location. A robotic approach with superior agility and precise movements of the robotic arms could provide the surgeon with better exposure and greater ergonomic comfort during the dissection of small anatomical structures, such as the pelvic cavity (2). In recent years, with the renewal of rectal cancer surgery and the advancements in equipment and surgical techniques, the anus preservation rate has been increasing. Many patients have avoided permanent stoma damage, and their quality of life has improved. There are growing anus preservation practices and new surgical methods, such as robots and natural orifice specimen extraction surgery (NOSES), in colorectal surgery. However, these new procedures could increase the risks inherent to complications, such as anastomotic leakage and damage to the autonomic nerve function of the pelvic floor, which adversely affect surgical outcomes and delayed hospital stay. Although the negative impact of complications on short-term effects is overtly clear, the impact on cancer recurrence and survival remains uncertain. A previous report indicated greater local recurrence risk and worse overall and cancer-specific survival in patients with anastomotic leakage (3). Other authors have reported alternative findings with no long-term impact on oncological outcomes in patients with complication (4). Our study retrospectively analyzed the clinical data of 641 cases of robotic rectal surgery in the mid- and low rectum in our hospital from January 2014 to December 2018. We explored the risk factors for complications and anastomotic leakage and their influence on long-term oncological outcomes.

## Materials and Methods

From January 2014 to December 2018, 641 cases of mid and low rectal cancer with robotic surgery were collected retrospectively at Zhongshan Hospital Fudan University. Inclusion criteria: a) 18–80 years old; b) preoperative biopsy confirmed adenocarcinoma; c) tumor within 10 cm from the anus; and d) standard radical surgery, including AR, LAR, and the NOSES procedure, with some undergoing stoma diversion. The exclusion criteria were as follows: a) the previous history of colorectal cancer surgery; b) tumor accompanied by distant metastases or other tumors; c) emergencies and abdominal adhesions, which precluded minimally invasive surgery; and d) combined organ resection.

The Fudan University Ethics Committee approved the study, and all patients provided informed consent. Preoperative data, pathological results, and complication information were obtained from electronic medical records, and postoperative survival information was collected during regular follow-ups.

### Surgical Approach

All of the patients underwent surgery using the da Vinci Surgical System. The TME was performed robotically in all of the patients. Anterior resection, low anterior resection, and the NOSES procedure were performed using the double-stapled technique. The NOSES procedure is shown in **Supplement S1** ****.

Intraoperative frozen pathological diagnosis was carried out using tissue from distal margins. The pelvic peritoneum was routinely closed. The methylene blue perfusion test was used to check anastomotic integrity. If blue dye leakage was found, we sutured the leakage point with 3-0 Vicryl followed by a retest of leakage using methylene blue. In some cases, the leakage persisted; we then performed prophylactic stoma diversion. In other cases, where the anastomosis was too low to suture, or the patients had severe tissue edema, prophylactic stoma diversion was also performed. A double cannula was placed near the anastomosis. When anastomotic leakage occurred, the cannula could be used to wash and drain and, in some cases, to avoid a salvage stoma.

### Complications and Anastomotic Leakage

Complications were diagnosed and categorized according to patients’ symptoms, with laboratory and radiological evaluations to confirm clinical suspicions. The grading of complications was scored based on the detailed tables of the Surgical Complications Severity Scoring System proposed by Mazeh et al. (5).

Anastomotic leakage after anterior resection of the rectum was defined as a defect of the intestinal wall integrity at the colorectal or colo-anal anastomotic site (including suture and staple lines of anorectal reservoirs) leading to communication between the intra- and extraluminal compartments. A pelvic abscess close to the anastomosis is also considered anastomotic leakage. Severity grading was as follows: Grade A, requiring no active therapeutic intervention; Grade B, requiring active therapeutic intervention but manageable without relaparotomy; and Grade C, requiring relaparotomy (6). Aspiration drainage or transverse colon stoma was performed according to the severity of the infection.

### Statistical Analysis

Statistical analysis was conducted using IBM SPSS Statistics software version 19 (SPSS Inc., IBM, Chicago, IL, USA). Categorical variables were analyzed using the χ2 test, and continuous variables were analyzed using the t-test. One-way analysis of variance was used for the study of quantitative differences between various groups. Logistic regression was used for multivariate analysis. The Kaplan–Meier method was used to calculate disease-free survival and overall survival. P values less than 0.05 were considered statistically significant.

## Results

### Clinical Data

**Figure 1:**
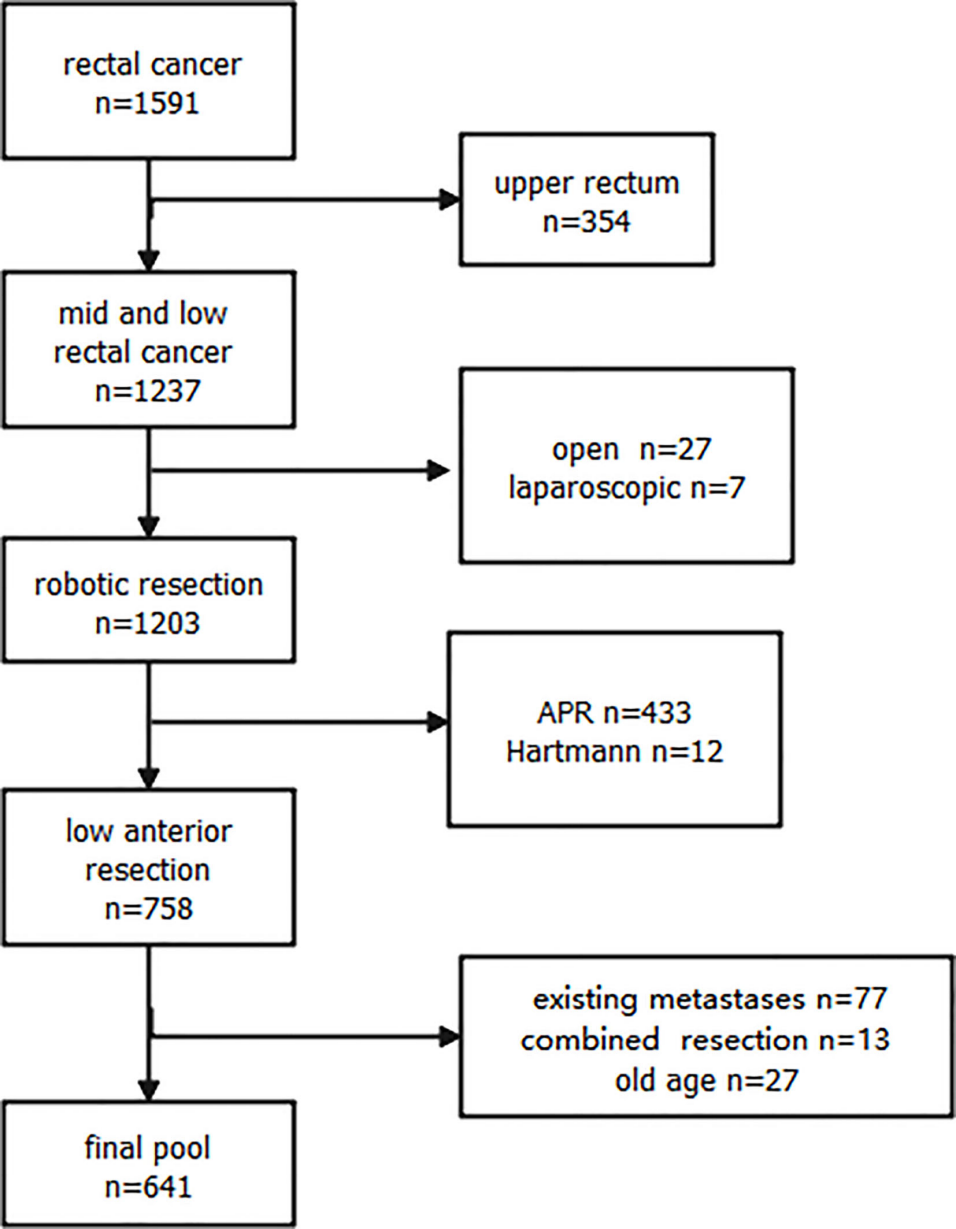
Flow Chart of Enrollment of Mid and Low Robotic Cancer.

The selection of patients included in the study is illustrated in **Figure 1**. A total of 1,591 patients were admitted with rectal cancer from 2014 to 2018, and 641 patients underwent robotic treatment in the mid and low rectum enrolled in the analyses.

Among the study patients, 403 cases were males (62.9%), and the average age was 60.9 ± 10.4 years. The median body mass index (BMI) was 23.4. A total of 53 (8.3%) patients were considered to have American Society of Anesthesiologists scores (ASA) of III. Anterior resection (AR) and low anterior resection (LAR) were performed in 516 (80.5%) patients, while 125 (19.5%) patients underwent the NOSES procedure. The mean operation time was 164.5 ± 47.5 min, and the mean estimated blood loss was 66.1 ± 35.9 ml. Intraoperative methylene blue leakage was found in 41 cases (6.4%). Diverting ileostomies were performed in 15 cases (2.3%). A total of 67 (10.5%) patients with T4 or N2 mid and low rectal cancer underwent preoperative radiochemotherapy. The mean duration of hospital stay after surgery was 7.73 ± 3.24 days. The mean number of harvested lymph nodes was 17.2 ± 6.7. The positive rate of circumferential margin (CRM) was 7 (1.1%). According to the American Joint Committee on Cancer (AJCC) TNM stage, stages I, II, and III patients accounted for 180 (28.1%), 191 (29.9%), and 270 (42.1%) patients, respectively. The characteristics of all patients are shown in **Supplement S2**.

### Surgical Complications

One hundred seventeen patients (17.6%) experienced surgical complications, with Grades 1–2 and Grades 3–4 complications accounting for 16.5% and 1.8%, respectively (5). There was no perioperative mortality. Anastomotic leakage occurred in 44 patients (6.9%), with one case of rectovaginal fistula. Based on severity grading, 16 (36.4%), 21 (47.7%), and 7 (15.9%) had Grade A, B, and C leakages, respectively. Two postoperative anastomotic leakages occurred in patients with a positive methylene blue test intraoperatively (2/41, 4.8%). Eleven cases had reoperation (1.7%), including seven cases of anastomosis leakage (1.1%), two cases of bleeding after surgery, and two cases of ileus. (**Table 1**). The mean postoperative hospital stay with complication was much longer than the hospital stay without complication (10.4 ± 5.6 vs. 7.1 ± 2.0 days, p<0.01). The mean postoperative hospital stay with leakage was much longer than that in the nonleakage group (12.5 ± 7.0 vs. 7.4 ± 2.4 days, P<0.01).

**Table 1:** Surgical complications.

Characteristics	Value; n(%)
Total complication rate^a^	117(18.3%)
Grades 1–2	106(16.5%)
Grade 3	10(1.6%)
Grade 4	1(0.2%)
Complications	
Infection events^b^	24(3.7%)
Anastomosis leakage	44(6.9%)
Urinary retention	18(2.8%)
Postoperative bleeding	5(0.8%)
Ileus	10(1.6%)
Gastric motility disorders	4(0.6%)
Organ dysfunction	4(0.6%)
Thrombotic events	2(0.3%)
Others	4(0.6%)
Mortality	0
Reoperation rate^c^	11(1.7%)

a Surgical complication rate and mortality analysis within 30 days of operation following the Mazeh system.

b Infection events included intraabdominal infection or abscess, catheter-derived infection, wound infection or lung infection, excluding anastomotic leakage events.

c The rates of reoperation related to surgical complications were analyzed within 90 days of operation.

### Long-Term Outcomes

**Figure 2:**
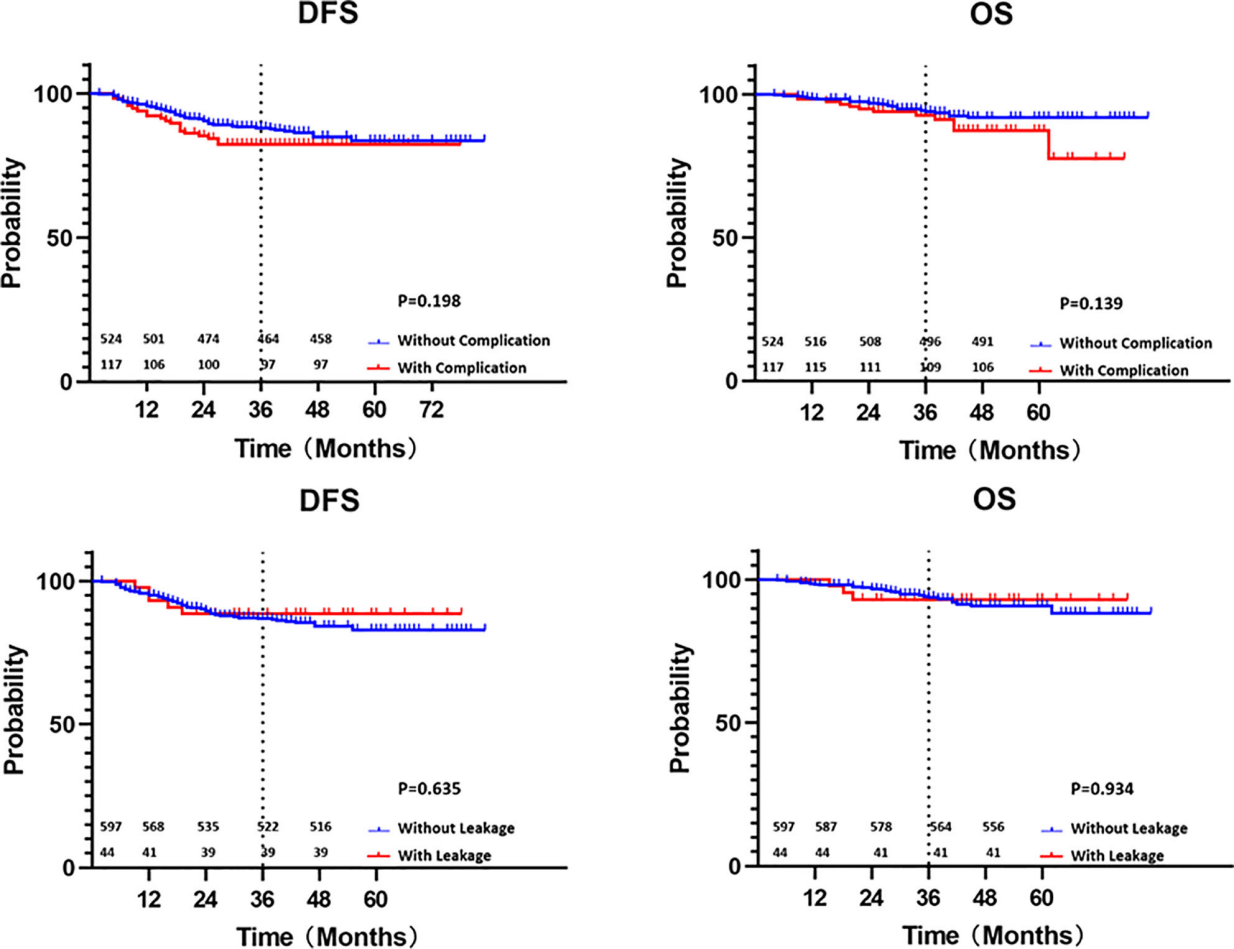
Kaplan-Meier curves of disease-free survival and overall survival with or without complication and leakage (survival numbers were added).

The median follow-up duration from primary treatment was 39 months (25th–75th percentile 30-47). Local recurrence occurred in 13 patients (2.0%), while distant metastases occurred in 87 patients (13.6%). The most common metastatic sites were the liver (5.1%) and lung (5.0%). Complications and anastomotic leakage showed no significant influences on local recurrences and distant metastases (**Table 2**). The 3-year disease-free survival rate of all cases was 87.1%, with rates of 82.4% with complication and 88.4% without complication (p=0.198) and 88.6% with leakage and 87.0% without leakage (P=0.635). The 3-year overall survival rate was 93.7%, with rates of 92.6% with complication and 94.0% without complication (p=0.139) and 93.1% with leakage and 93.8% without leakage (P=0.934) (**Figure 2**).

**Table 2:** Recurrence patterns with complication and anastomotic leakage.

Outcomes	Total N=641		Complications			Leakage		
			(+)n=117	(-)n=524	P	(+)n=44	(-)n=597	P
Local recurrence		13(2.0%)	2(1.7%)	11(2.1%)	0.787	1(2.2%)	12(2.0%)	0.905
Metastases^a^		74(11.5%)	18(15.4%)	56(10.7%)	0.150	4(9.0%)	70(11.7%)	0.598
Liver		33(5.1%)						
Lung		32(5.0%)						
Bone		4(0.6%)						
Peritoneal		11(1.7%)						
Brain		2(0.3%)						
Others^b^		6(0.9%)						

a Some patients had multi-site metastases.

b Others include two cases of abdominal wall, two cases of the spleen, one case of the ovary, and one case of the adrenal gland.

### Risk Factors Associated With Complication

Univariate analysis showed that male sex, ASA score, tumor distance within 5 cm from the anus, prolonged operation time, and prophylactic stoma diversion were associated with overall complications after robotic mid and low rectal cancer surgery (P<0.05). Preoperative radiotherapy and the NOSES procedure showed no significant effect on complications in our study.

Multivariate analyses showed that male sex (OR=1.855, 95% CI: 1.175–2.923, P<0.05), a tumor distance of 5 cm from the anus (OR=1.563, 95% CI: 1.016–2.404, P<0.05), and a prolonged operation time (OR=1.563, 95% CI: 1.009–2.421, P<0.05) were independent risk factors for complications after robotic mid and low rectal procedures (**Table 3**).

**Table 3:** Risk factors associated with complication.

Clinical factors	N	with complication (%)	Univariate analysis		Multivariate analysis	
			*OR* value (95% *CI*)	P-value	OR value (95% CI)	P-value
Gender			1.812 (1.159–2.833)	0.009*	1.855 (1.175–2.923)	0.008*
Feale	238	31(13.0%)				
Male	403	86(21.3%)				
Age			0.973 (0.646–1.464)	0.894		
≥65	254	47(18.5%)				
<65	387	70(18.1%)				
BMI(kg/m^2^)			1.033 (0.671–1.591)	0.881		
≥25	199	37(18.6%)				
<25	442	80(18.1%)				
ASA score			1.444 (1.057–1.973)	0.021*		
I–II	588	101 (17.2%)				
III	53	16 (18.9%)				
Diabetes			1.307 (0.742–2.302)	0.354		
yes	82	18(22.0%)				
no	559	99(17.7%)				
Hb(g/L)			1.182 (0.644–2.168)	0.590		
<110	73	15(20.5%)				
≥110	528	102(19.3%)				
ALB(g/L)			1.220 (0.791–1.884)	0.369		
<40	181	37(20.4%)				
≥40	460	80(17.4%)				
CEA (ng/ml)			1.162 (0.767–1.760)	0.479		
<5	418	73(17.5%)				
≥5	223	44(19.7%)				
Tumor location from anus(cm)			1.562 (1.025–2.387)	0.038*	1.563 (1.016–2.404)	0.042*
>5	456	74(16.2%)				
≤5	185	43(23.2%)				
Tumor size(cm)			1.242 (0.830–1.858)	0.293		
>5	268	54(20.1%)				
≤5	373	63(16.9%)				
preoperative radiotherapy			0.975 (0.504–1.884)	0.939		
yes	67	12(17.9%)				
no	574	105(18.3%)				
Operation			1.152 (0.704–1.886)	0.573		
NOSES	125	25(20.0%)				
AR or LAR	516	92(17.8%)				
Diverting stoma			3.093 (1.079–8.867)	0.036*		
yes	15	6(40.0%)				
no	626	111(17.7%)				
Estimated blood loss(ml)			1.140 (0.702–1.853)	0.597		
≥100	131	26(19.8%)				
<100	510	91(17.8%)				
Operation time(min)			1.616 (1.065–2.451)	0.024*	1.563 (1.009–2.421)	0.046*
<180	445	71(16.0%)				
≥180	196	46(23.5%)				
Pathological type			1.265 (0.731–2.189)	0.400		
adenocarcinoma	525	99(18.9%)				
Mucinous adenocarcinoma	116	18(15.5%)				
Differentiation			1.105 (0.904–1.351)	0.329		
I–II	322	54(16.8%)				
III–IV	319	63(19.7%)				
Vascular invasion			1.089 (0.687–1.727)	0.716		
yes	156	30(19.2%)				
no	485	87(17.9%)				
Perineural invasion			1.084 (0.699–1.682)	0.718		
yes	183	35(19.1%)				
no	458	82(17.9%)				
N stage			0.727 (0.480–1.101)	0.132		
yes	270	42(15.6%)				
no	371	75(20.2%)				
T stage			0.906 (0.737–1.114)	0.350		
T_1˜2_	228	46(20.2%)				
T_3˜4_	413	71(17.2%)				

BMI, body mass index; ALB, albumin; ASA, American Society of Anesthesiologists; AR, anterior resection; LAR, low anterior resection; NOSES, natural orifice specimen extraction surgery.

*P < 0.05.

### Risk Factors Associated With Anastomotic Leakage

Univariate and multivariate analyses showed similar results: being male (OR=2.247, 95% CI: 1.126–4.902, P<0.05), a tumor distance of 5 cm from the anus (OR= 2.242, 95% CI: 1.197–4.202, P<0.05), and operation time length (OR= 2.114, 95% CI: 1.127–3.968, P<0.05) were significantly associated with the occurrence of anastomotic leakage after robotic mid and low rectal cancer surgery. Preoperative radiotherapy, stoma diversion, and the NOSES procedure showed no significant effect on anastomosis leakage in our study (**Table 4**).

**Table 4:** Risk factors associated with anastomosis leakage.

Clinical factors	Cases	Leakage cases (%)	Univariate analysis		Multivariate analysis	
			*OR* value (95% *CI*)	P-value	OR value (95% *CI*)	P-value
Gender			2.101 (1.018–4.329)	0.044*	2.247 (1.126–4.902)	0.023*
Female	238	10(4.2%)				
Male	403	34 (11.3%)				
Age			1.160 (0.614–2.191)	0.647		
≥65	254	16(6.3%)				
<65	387	28(7.2%)				
BMI(kg/m^2^)			1.161 (0.608–2.217)	0.651		
≥25	199	15(7.5%)				
<25	442	29(6.6%)				
ASA score			1.058 (0.620–1.804)	0.837		
I–II	588	40 (6.8%)				
III	53	4 (7.5%)				
Diabetes			1.082 (0.443–2.646)	0.862		
yes	82	6(7.3%)				
no	559	38(6.8%)				
Hb(g/L)			0.765 (0.266–2.204)	0.620		
<110	73	4(5.5%)				
≥110	528	40(7.6%)				
ALB(g/L)			1.496 (0.789–2.837)	0.217		
<40	181	16(8.8%)				
≥40	460	28(6.1%)				
CEA (ng/ml)			0.866 (0.449–1.670)	0.668		
<5	418	30(7.2%)				
≥5	223	14(6.3%)				
Tumor location from anus(cm)			2.183 (1.174–4.049)	0.014*	2.242 (1.197–4.202)	0.012*
>5	456	24(5.3%)				
≤5	185	20(10.8%)				
Tumor size(cm)			0.782 (0.414–1.477)	0.449		
>5	268	16(6.0%)				
≤5	373	28(7.5%)				
preoperative radiotherapy			1.106 (0.420–2.911)	0.838		
yes	67	5(7.4%)				
no	574	39(6.8%)				
Operation			1.066 (0.499–2.280)	0.869		
NOSES	125	9(7.2%)				
AR or LAR	516	35(6.8%)				
Diverting stoma			0.968 (0.124–7.540)	0.976		
yes	15	1(6.6%)				
no	626	43(6.9%)				
Estimated blood loss(ml)			1.702 (0.864–3.354)	0.124		
≥100	131	13(9.9%)				
<100	510	31(6.1%)				
Operation time(min)			1.992 (1.073–3.704)	0.029*	2.114 (1.127–3.968)	0.020*
<180	445	24(5.4%)				
≥180	196	20 (10.2%)				
Pathological type			1.181 (0.513–2.719)	0.696		
adenocarcinoma	525	37(7.0%)				
Mucinous adenocarcinoma	116	7(6.0%)				
Differentiation			0.957 (0.704–1.300)	0.779		
I–II	322	23(7.1%)				
III–IV	319	21(6.6%)				
Vascular invasion			0.787 (0.370–1.676)	0.535		
yes	156	9(5.8%)				
no	485	35(7.2%)				
Perineural invasion			1.321 (0.691–2.526)	0.400		
yes	183	15(8.2%)				
no	458	29(6.3%)				
N stage			0.772 (0.409–1.457)	0.424		
yes	270	16(5.9%)				
no	371	28(7.5%)				
T stage			0.842 (0.618–1.148)	0.276		
T_1˜2_	228	19(8.3%)				
T_3˜4_	413	24(5.8%)				

BMI, body mass index; ALB, albumin; ASA, American Society of Anesthesiologists; AR, anterior resection; LAR, low anterior resection; NOSES, natural orifice specimen extraction surgery.

*P < 0.05.

## Discussion

With the rise of minimally invasive surgery, radical resection of mid and low rectal cancer has increased, with the goal of causing less trauma and pursuing better functional preservation. Postoperative complications could prolong hospitalization, increase the medical burden, and decrease quality of life, which are among the challenging problems faced by mid and low rectal cancer surgery (7). Due to the narrow area of the pelvis and the limited operation space, traditional laparoscopic rectal cancer operations have disadvantages, such as operative difficulty, increased requirements for assistants, and a steep learning curve (8). Some studies have reported no difference in the incidence of complications between robotic surgery and traditional laparoscopic surgery (9). Since 2010, the robotic surgery system has been gradually applied to radical surgery for mid and low rectal cancer due to its capability of producing high-definition images and its stable and flexible operation (10). These features are advantageous in distinguishing pelvic nerves, dissecting the anterior and lateral tissues, and suturing.

Anastomotic leakage is one of the most critical complications postoperatively. Many factors may affect anastomotic leakage after rectal cancer surgery. Studies have shown that being male, smoking, diabetes, hypoalbuminemia, and obesity are potentially high-risk factors for anastomotic leakage (11–13). Frouws’ analysis (14) showed that men are at high risk for anastomotic Grade C leakage in patients with low rectal cancer. In this study, being male was an independent risk factor for complications and leakage. Five men and two women underwent reoperations for anastomotic leakage in the cohort. One of the women suffered from a rectovaginal fistula. This could be explained by the fact that the male pelvic outlet is narrow and difficult to operate, resulting in an unsatisfactory anastomosis. The ASA score is a subjective assessment of patients’ overall health. A high ASA score might lead to high risks of complications after surgery. In our study, a high score was associated with more complications but was not an independent factor. Conversely, preoperative smoking cessation, respiratory management, strict blood sugar control, and nutritional support are protective factors for reducing postoperative complications.

In our study, we observed that a short distance between the tumor and the anal margin was associated with a higher incidence of complications and anastomotic leakage. Multivariate analysis confirmed that the distance from the anal margin is an independent risk factor for complications and anastomotic leakage. The reasons could be the difficulty of dissections and anastomosis, the inability to suture in a limited space, and insufficient blood supply. Fukada’s research (11) showed that tumors less than 6 cm from the anal margin were correlated with increased anastomotic leakage after rectal cancer.

Yasui et al. (15) reported that a tumor diameter larger than 4 cm was a risk factor for anastomotic leakage after rectal cancer surgery. However, there was no apparent correlation between tumor size and complications or anastomotic leakage in our study. As laparoscopic and robotic surgery for rectal cancer is associated with attenuated blood loss, this technique appears beneficial compared with an open approach (16). Leichtle (17) reported that blood loss greater than 100 ml is associated with an increased risk of complications. In our study, blood loss was not associated with higher complication or leakage rates.

Neoadjuvant chemoradiotherapy is beneficial to tumor regression and preoperative downstaging of advanced mid and low rectal cancer. With this treatment, some patients would have the opportunity to save the anus and reduce the local recurrence rate of tumors after surgery. Schiffman et al. (18) reported that the incidence of postoperative anastomotic leakage was 26.6% in patients who received preoperative chemoradiation, while the incidence of patients who did not receive chemoradiation was only 9.7%. Neoadjuvant chemoradiotherapy may cause tissue edema, which increases the difficulty of surgery, accompanied by vascular endothelial degeneration. Moreover, it increases the probability of microthrombosis and affects the blood supply of tissues around the anastomosis, leading to increased leakage. Borstlap et al. (19) analyzed the data of 2095 patients and found that neoadjuvant chemoradiotherapy was an independent risk factor for anastomotic leakage. In Borstlap’s study, anastomotic leakage was diagnosed in 13.4% of the patients within 30 days, with almost routine use of neoadjuvant radiotherapy (88.8%) in the patients. Frouws et al. (14) indicated that patients with neoadjuvant therapy were at risk for increased postoperative Grade B anastomotic leakage. However, the controversy remains. Randomized controlled studies from Sebag-Montefiore (20) suggested that neoadjuvant chemoradiation did not increase the incidence of anastomotic leakage. Salmenkylä et al. (21) also showed that the anastomotic leakage rate did not significantly differ between the surgery and radiochemotherapy groups (20.6% vs. 27.4%). In this study, neoadjuvant chemoradiation did not show a significant increase in the risk of complications and leakage. The incidence of anastomotic leakage after preoperative radiochemotherapy was 7.4%, compared with 6.8% in the surgery group.

In recent years, natural orifice specimen extraction surgery (NOSES) has been gradually applied in the treatment of colorectal cancer (22). The abdominal incision to extract the specimen is avoided, and the ultimate cosmetic effect is achieved with radical treatment. The NOSES procedure should reduce postoperative pain, surgical wound infection, and incisional hernia (23). Ding’s study (24) showed that the NOSES rectal cancer procedure did not increase short-term complications and may reduce intraoperative bleeding and patient pain. Liu et al. (25) reported that the NOSES rectal cancer procedure did not increase the occurrence of short-term complications such as anastomotic leakage. In our study, 127 cases of robotic NOSES in the mid and low rectum did not increase the incidence of postoperative complications. The incidence rates of complications and anastomotic leakage were 20.0% and 7.2% in the NOSES procedure, respectively, which seemed higher than those in the LAR group but showed no significant difference.

It should be noted that this study reflects single-center data, and both the surgeon and assistant have rich experience in robotic rectal cancer surgery. A prolonged operation time might indicate that the operation is more complicated. Antonio’s study (26) showed that a longer operating time was significantly associated with leakage among the operative factors. In our study, prolonged operation time was also significantly correlated with increasing incidence rates of complications and anastomotic leakage. We thus suggest that when facing difficulty in dissection or hemostasis, timely transfer of the patient to open surgery or stoma diversion could be considered.

Maeda et al. (27) reported the efficacy of intracorporeal reinforcing sutures for anastomotic leakage after laparoscopic surgery for rectal cancer. The methylene blue perfusion test was used to check the defective location. The number of linear stapler firings during rectal division is associated with anastomotic leakage. Lateral intersecting staple lines (dog-ears) are a weak point in the anastomosis (28), and intersections of staple lines after the double stapling technique tend to be associated with anastomotic leakage (29). Anastomotic reinforcing sutures were used in the defective parts and weak points. In our study, the pelvic peritoneum was routinely closed to reduce the risks of diffuse peritonitis, which made washing and drainage in the pelvic cavity possible. Seven cases (1.1%) of reoperation occurred in 44 cases of anastomotic leakage in this study.

It is still controversial whether a stoma diversion is necessary for mid and low rectal cancer operations. A meta-analysis (30) with 44.5% of patients undergoing a stoma diversion suggested that a stoma diversion could reduce the incidence of serious complications such as peritonitis caused by anastomotic leakage, as well as the reoperation rate and mortality. However, a stoma diversion may cause many inconveniences to patients. Garfinkle et al. (31) reported that 8% of patients had complications such as intestinal obstruction after loop ileostomy closure. Song et al. (32) reported that in 520 cases of loop ileostomy closure, 9.8% of patients underwent restoma due to postoperative complications. The reasons may include anastomosis-related complications, local recurrence, and loss of anal sphincter function. In this study, 15 patients (2.3%) underwent prophylactic ileostomy. Stoma diversion showed more complications after surgery but was not an independent risk factor. Who would benefit from a stoma diversion is still a question. In Shimizu’s study (33), ileostomy diversion following laparoscopic LAR decreased the risk of anastomotic leakage, especially in male patients with malnutrition, and due to ileostomy diversion-related morbidity, the procedure was not recommended in female patients. Kim et al. (34) reported that ileostomy diversion could increase the risk of anastomotic stricture. Anastomotic stenosis more easily occurs in patients with stoma diversion. Regular expansion and functional exercise of the anus is recommended after the anastomosis is healed.

Pinar et al. (35) reported that robotic surgery was equivalent to conventional laparoscopy in rectal cancer patients. The 3-year DFS and OS in our cohort were 87.1% and 93.7%, respectively, which were similar to other studies evaluating laparoscopic surgery for mid and low rectal cancer (36). Complications and anastomotic leakage showed no significant influences on the long-term outcomes, which was similar to some reports (4). There seemed to be a negative trend with complication in the long-term results in our study, but the difference was not significant. Thus, further research is needed. Care should be taken to reduce the instance of complications, including anastomotic leakage and surgical duration, by optimizing the operating team (37).

Limitations: Single-institution experiences with no direct comparison to a laparoscopic or open group are not adequate to make universal conclusions, which is the major limitation of our study. In addition, as this is a single-center, retrospective study, the surgeon’s background may affect the results to some extent. There is the possibility that the patients may have been a highly selected group with good clinical status. Therefore, we must consider that selection bias may have played a role in our results. Furthermore, it is possible that longer surgical times and more blood loss are simply markers of surgical difficulty.

## Conclusion

Robotic rectal resection was safe and adequate in treating mid and low rectal tumors in our study. Being male, having a lower tumor location, and having a prolonged operation time were independent risk factors for complication and anastomotic leakage in mid and low rectal cancer. The complication and anastomotic leakage might have no long-term impact on oncological outcomes for mid and low rectal cancer with robotic surgery.

## Data Availability Statement

The raw data supporting the conclusions of this article will be made available by the authors, without undue reservation.

## Ethics Statement

The studies involving human participants were reviewed and approved by The Fudan University Ethics Committee. The patients/participants provided their written informed consent to participate in this study.

## Author Contributions

JC, ZZ, WC, and TY contributed equally to this work data collection and analysis. QF and DZ helped data collection. YW and GH guided the study. All authors contributed to the article and approved the submitted version.

## Funding

This project was supported by The National Natural Science Foundation of China (81472228), Shanghai Science and Technology Committee Project (19140900703), National Natural Science Foundation of China (82072652), and National Key Research and Development Program of China (2017YFC0908205).

## Conflict of Interest

The authors declare that the research was conducted in the absence of any commercial or financial relationships that could be construed as a potential conflict of interest.
