# Supplementary material for: Short-Term and Long-Term Outcomes in Mid and Low Rectal Cancer With Robotic Surgery
Source: Front Oncol. 2021 Mar 9;11:603073. doi: 10.3389/fonc.2021.603073 (PMC7985529; doi:10.3389/fonc.2021.603073)
Supplement: Supplementary file 1 [file DataSheet_1.docx]

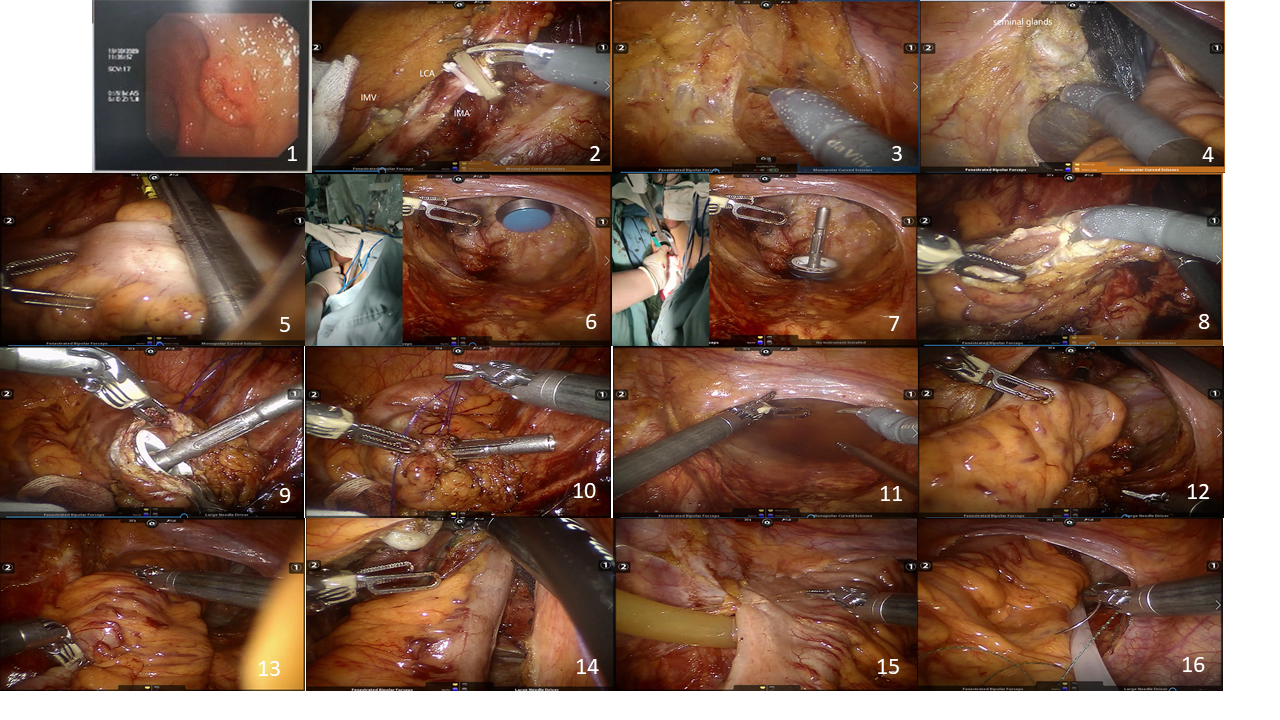


Supplement Figure S1: The Procedure of NOSES in Our Center

A 44-year-old male had a BMI of 19.9, a tumor size of 2 cm, and a tumor location 5 cm from the anus. Nanocarbon lymphatic tracer was injected before surgery; the rectum turned black around the tumor area in this case.

(1) Rectal cancer, as shown by coloscopy

(2) Lymph node dissection around the IMA, preserving the roots of the IMA and the LCA

(3) Separation of the posterior part of pararectal space

(4) Separation of the anterior and lateral parts of the pararectal space

(5) Cutting of the rectum with a stapler

(6) Extraction of the specimen from the anus and placement of the head of the stapler into the pelvic cavity.

(7) Cutting open the distal rectum with a stapler

(8) Opening the proximal colon

(9) Placing the head of the stapler into the proximal colon

(10) Purse-string suture and fixation of the head of the stapler

(11) Rinsing of the pelvic cavity

(12) Anastomosis

(13) Methylene blue perfusion test

(14) Suturing of the anastomosis

(15) Closure of the pelvic peritoneum and placement of a cannula on the left side of the anastomosis.

(16) Closure of the pelvic peritoneum and placement of another cannula on the right side of the anastomosis.
