# Supplementary material for: Short-Term and Long-Term Outcomes in Mid and Low Rectal Cancer With Robotic Surgery
Source: Front Oncol. 2021 Mar 9;11:603073. doi: 10.3389/fonc.2021.603073 (PMC7985529; doi:10.3389/fonc.2021.603073)
Supplement: Supplementary file 2 [file Table_1.docx]

**Table S1** Characteristic of 641 cases of mid and low robotic rectal surgery

| Clinical factors | Cases | percentage（%） | mean ±SD（range） |  |  |
| --- | --- | --- | --- | --- | --- |
| Gender |  |  |  |  |  |
| Male | 403 | 62.9% |  |  |  |
| Female | 238 | 37.1% |  |  |  |
| Age（year） |  |  | 60.9±10.4(25-80) |  |  |
| ≥65 | 254 | 39.6% |  |  |  |
| <65 | 387 | 60.4% |  |  |  |
| BMI（kg/m^2^） |  |  |  |  |  |
| ≥25 | 199 | 31.0% |  |  |  |
| <25 | 442 | 69.0% |  |  |  |
| ASA score |  |  |  |  |  |
| I-II | 588 | 91.7% |  |  |  |
| III | 53 | 8.3% |  |  |  |
| Diabetes |  |  |  |  |  |
| yes | 82 | 12.8% |  |  |  |
| no | 559 | 87.2% |  |  |  |
| Hb（g/L） |  |  |  |  |  |
| ＜110 | 73 | 11.4% |  |  |  |
| ≥110 | 568 | 88.6% |  |  |  |
| ALB（g/L） |  |  |  |  |  |
| <40 | 181 | 28.2% |  |  |  |
| ≥40 | 460 | 71.8% |  |  |  |
| CEA (ng/ml) |  |  |  |  |  |
| <5 | 418 | 65.2% |  |  |  |
| ≥5 | 223 | 34.8% |  |  |  |
| Tumor location from anus（cm） |  |  |  |  |  |
| >5 | 456 | 71.1% |  |  |  |
| ≤5 | 185 | 28.9% |  |  |  |
| Tumor size（cm） |  |  |  |  |  |
| >5 | 268 | 41.8% |  |  |  |
| ≤5 | 373 | 58.2% |  |  |  |
| preoperative radiotherapy^a^ |  |  |  |  |  |
| yes | 67 | 10.5% |  |  |  |
| no | 574 | 89.5% |  |  |  |
| preoperative chemotherapy or CRT^b^ |  |  |  |  |  |
| yes | 313 | 48.8% |  |  |  |
| no | 328 | 51.2% |  |  |  |
| Operation performed |  |  |  |  |  |
| NOSES | 125 | 19.5% |  |  |  |
| AR or LAR | 516 | 80.5% |  |  |  |
| Diverting stoma |  |  |  |  |  |
| yes | 626 | 97.7% |  |  |  |
| no | 15 | 2.3% |  |  |  |
| Estimated blood loss（ml） |  |  | 66.1±35.9(20-400) |  |  |
| ≥100 | 131 | 20.4% |  |  |  |
| <100 | 510 | 79.6% |  |  |  |
| Operation time（min） |  |  | 164.5±47.5(57-420) |  |  |
| <180 | 445 | 69.4% |  |  |  |
| ≥180 | 196 | 30.6% |  |  |  |
| Pathological type |  |  |  |  |  |
| adenocarcinoma | 525 | 81.9% |  |  |  |
| Mucinous adenocarcinoma | 110 | 17.2% |  |  |  |
| other | 6 | 0.9% |  |  |  |
| Differentiation |  |  |  |  |  |
| I | 12 | 1.9% |  |  |  |
| II | 310 | 48.4% |  |  |  |
| III | 296 | 46.2% |  |  |  |
| other | 6 | 0.9% |  |  |  |
| Vascular invasion |  |  |  |  |  |
| yes | 485 | 75.7% |  |  |  |
| no | 156 | 24.3% |  |  |  |
| Perineural invasion |  |  |  |  |  |
| yes | 458 | 71.5% |  |  |  |
| no | 183 | 28.5% |  |  |  |
| N stage |  |  |  |  |  |
| N_0_ | 371 | 57.9% |  |  |  |
| N_1-2_ | 270 | 42.1% |  |  |  |
| T stage |  |  |  |  |  |
| T_1_ | 66 | 10.3% |  |  |  |
| T_2_ | 166 | 25.9% |  |  |  |
| T_3_ | 360 | 56.2% |  |  |  |
| T_4_ | 49 | 7.6% |  |  |  |
| AJCC |  |  |  |  |  |
| I | 180 | 28.1% |  |  |  |
| II | 191 | 29.8% |  |  |  |
| III | 270 | 42.1% |  |  |  |
| Postoperative hospital stay (days) |  |  | 7.73±3.24 (5-37) |  |  |

BMI body mass index, ALB albumin, ASA American Society of Anesthesiologists, AR anterior resection, LAR low anterior resection, NOSES natural orifice specimen extraction surgery, AJCC stage indicates the American Joint Committee on Cancer TNM classification.

a Patients with T4 or N2 disease accepted radiotherapy or chemoradiotherapy.

b Patients with postoperative pathology TNM stage with III stage or high-risk II stage, received chemotherapy or CRT
